# How Glucagon-Like Peptide-1 Medications Are Depicted in Instagram Posts Regarding Women’s Health, Nontraditional Access, and Barriers to Access: Content Analysis

**DOI:** 10.2196/66400

**Published:** 2025-09-04

**Authors:** Brittnie E Bloom, Marie A Bragg, Melanie R Jay, Daphna Harel, Camile Cline, Matthew Crowe, Avery Montoya, Sandhya Muthuramalingam, Roberto Santana, Stephanie L Albert

**Affiliations:** 1 School of Public Health College of Health and Human Services San Diego State University San Diego, CA United States; 2 Department of Population Health New York University Grossman School of Medicine New York, NY United States; 3 Department of Medicine NYU Grossman School of Medicine New York, NY United States; 4 New York Harbor Veterans Affairs Healthcare System New York, NY United States; 5 Applied Statistics, Social Science, and Humanities Steinhardt School of Culture, Education, and Human Development New York University New York, NY United States

**Keywords:** glucagon-like peptide-1 medications, social media, polycystic ovarian syndrome, women’s health, access to care

## Abstract

**Background:**

Glucagon-like peptide-1 (GLP-1) medications, recently introduced in the United States for treating type 2 diabetes and obesity, have sparked interest and discussion on social media. Social media has emerged as a prominent platform for the distribution of health information; its vast user base and accessibility make it a popular resource for individuals seeking medical advice. This study characterized GLP-1 medication–related content on Instagram about 3 critical areas of public health: women’s health, access from nontraditional settings, and barriers to access.

**Objective:**

This study aimed to perform passive content analysis in which information patterns would be observed from Instagram posts.

**Methods:**

We examined 40 GLP-1 medication–related Instagram posts to develop a list of the most frequently used hashtags. In total, 10 “top posts” were collected for 7 days (July 11-17, 2023) for 11 study hashtags (eg, #Ozempic). Duplicates, deleted posts or accounts, non-English content, and unrelated posts were removed. Each post was reviewed by at least 2 coders. Coding discrepancies were resolved through discussion.

**Results:**

The final sample included 239 posts. More than half of the posts (130/239, 54%) were from GLP-1 medication users. Raters perceived most users to be female (90/97, 92.8%); inferred that most used medications for weight loss (69/130, 53.1%); determined the most frequently noted health condition was polycystic ovarian syndrome (25/130, 19.2%); and judged posts to have positive sentiments about well-being (64/130, 49.2%) and toward the medications (100/130, 76.9%). About a quarter of the posts (55/239, 23%) offered services for obtaining GLP-1 medications; GLP-1 medications were perceived to be accessible via nontraditional health care settings (eg, medical spas) versus traditional settings (39/239,16.3% vs 12/239, 5%). Most users (78/97, 80.4%) were perceived to be White; barriers to access (ie, shortages, insurance, and cost) were infrequently mentioned (6/239, 2.5%; 3/239, 1.3%; and 1/239, 0.4%, respectively).

**Conclusions:**

Our findings highlight the perceived benefits of GLP-1 medications for women’s health, the need to increase health literacy about where to safely access medications, and how additional attention is needed for equitable access to GLP-1 medications. The onus is on social media companies to promote content that is safe and for the health care system and its payers to address health care inequities for historically marginalized communities.

## Introduction

Social media has emerged as a prominent platform for the distribution of health information; its vast user base and accessibility make it a popular resource for individuals seeking medical advice [1]. Social media platforms, such as Instagram, Facebook, and TikTok, have become increasingly influential sources of health information, shaping public opinion, attitudes, and behaviors on new and emerging trends in health and wellness [2] through user-generated content, influencers, and algorithm-driven exposure. With billions of active users worldwide, social media platforms offer a diverse array of health-related content, including information on diagnoses and treatment offered by its users, including social media influencers who may or may not have the appropriate credentials to provide advice on these topics [3]. Recently, social media platforms have played a significant role in shaping conversations and disseminating information about glucagon-like peptide-1 (GLP-1) or GLP-1 receptor agonist medications [4-6], a class of drugs that mimic the action of naturally occurring hormones that regulate blood sugar levels and the appetite. However, little is known about how social media users use and interact with social media platforms, specifically regarding new and emerging medications such as GLP-1 medications. Of note, for the remainder of the paper, we will use the term GLP-1 to describe this class of drugs.

GLP-1 medications were recently introduced to the US market with both great excitement and trepidation [7]. These drugs, including semaglutide (Ozempic and Wegovy) and tirzepatide (Mounjaro and Zepbound), have demonstrated substantial promise in addressing the root causes of 2 highly occurring chronic diseases: type 2 diabetes and overweight and obesity. For people with type 2 diabetes, GLP-1 medications can help improve glycemic control and reduce the risk of diabetes-related complications [8,9]. For those with overweight and obesity, GLP-1 medications can slow down gastric emptying and enhance the brain’s appetite control centers, leading to weight loss [10]. Given the success of GLP-1 medications and the myriad ways that the public is using the medications, the demand for them has become increasingly publicized—especially on social media platforms [11-13]. Social media platforms have provided a space where patients, “influencers,” health care professionals (including telemedicine-using health care providers), patient advocacy groups, and pharmaceutical companies alike can share their experiences, opinions, health outcomes, and related research findings on GLP-1 medications [14].

The use of GLP-1 medications is growing, both for US Food and Drug Administration (FDA)–approved and off-label use. Han et al [15] documented the increase in public interest in GLP-1 medications and how social media–related GLP-1 medication attention was associated with an increase in GLP-1 medication–related search terms. The GLP-1 medication information frenzy brought to light additional concerns including off-label use of the medication [15], medication shortages [16], the rise of counterfeit GLP-1 medications infiltrating the market from legitimate and illegitimate suppliers [17], and access to care. This is especially important considering the novelty and expense of GLP-1 medications [18-20] and the lack of coverage by insurance companies [21]. Furthermore, research continues to highlight the benefits of GLP-1 medications on health conditions outside of type 2 diabetes and overweight and obesity, such as polycystic ovarian syndrome (PCOS), endometriosis [22], and infertility [23]. Given the well-documented and persistent underrepresentation of women’s health issues in research [24], focusing on women’s use of GLP-1 medications and their associated health outcomes is needed and is a focal point of this research study.

Social media has the power to open up discussions related to GLP-1 medications that extend well-beyond the physician’s office; nevertheless, there is a paucity of research on the popular discourse around GLP-1 medications. Given the documented health benefits of these medications and public attention they receive, we sought to characterize social media posts about GLP-1 medications as they related to 3 critical areas of public health: women’s health, nontraditional access, and barriers to access. Our a priori expected observations (EOs) were as follows:

1. Posts will be dominated by female-presenting Instagram users and contain references specifically related to women’s physical and mental health.
2. Posts will offer services to consumers that facilitate access to GLP-1 medications from places perceived to be outside of traditional health care settings (eg, primary care, obesity medicine, endocrinology, or other specialty care).
3. Posts will address issues related to GLP-1 medication access, including equity in use among marginalized populations, shortages, coverage, and cost.

## Methods

### Ethical Considerations

This study used preexisting publicly available data and does not require institutional review board review per Federal Regulations for the Protection of Human Research Subjects (45 CFR 46.104 (d)(4)(i) [25]). Instagram users have the right to decide if they want their activity to be private and can do so by making their accounts private or limiting who can see their content. As such, this study only included posts, comments, and replies from public profiles that were part of the public domain.

### Hashtag Identification

We used a methodology to develop an empirically driven list of common hashtags used in GLP-1 medication–related social media posts, as has been done in previous research [26]. To do this, we chose 4 terms associated with the names of medications that had FDA approval for the treatment of diabetes or obesity and recorded how many Instagram posts contained each of those hashtags: #ozempic (71.5K), #semaglutide (53.9K), #wegovy (24.1K), and #mounjaro (20.9K). Using the 4 hashtags, we reviewed the first 10 “top posts” on Instagram (n=40) and documented all hashtags that appeared in those posts. The goal was to identify the 10 hashtags associated with the largest number of posts to serve as the study’s primary hashtags. Due to ties, 11 hashtags were selected for inclusion in this study: #ozempic (n=27), #weightloss (n=23), #semaglutide (n=21), #mounjaro (n=20), #weightlossjourney (n=19), #wegovy (n=17), #mounjaroweightloss (n=7), #ozempicweightloss (n=7), #health (n=6), #tirzepatide (n=6), and #wellness (n=6). The resulting empirically driven list includes hashtags related specifically to GLP-1 medications, weight loss, health, and wellness more generally.

### Selection of Instagram Posts

The objective of this study was to perform passive content analysis in which information patterns would be observed from Instagram posts. Similar to a study by Lazuka et al [27], we manually captured screenshots of the first 10 “top posts” on Instagram over a 1-week period (July 11-17, 2023) for each of the 11 hashtags (n=770). We also recorded metadata including links to the original post; date of post; information regarding the poster’s account (eg, number of followers); engagement metrics for each post (ie, number of likes, video views, and comments), and all captions and hashtags included in posts. When there was more than one picture in a post (ie, carousel photos), only the first photo was included in the study. Duplicates, deleted posts, deleted accounts, and non-English posts were removed from the sample, leaving 550 posts for coding. Our final sample exceeded the typical range in similar studies (ie, n=25 to n=500), suggesting that if posts were later excluded, we would still have a sufficient sample for thorough analysis [28].

### Codebook and Coding Procedures

We developed a codebook to guide the content analysis of Instagram posts based on our a priori EOs. For each code, we included a description of the code as well as detailed instructions of how the code should be applied to posts. Each post was assessed to determine if it was relevant to GLP-1 medications (yes vs no), what the post was about (a user, someone else, the medication more generally), and whether the post was an advertisement or paid partnership (yes vs no).

The codebook included the following codes for EO 1 about women’s health: observed gender of the poster (male [cis or transgender], female [cis or transgender], other, cannot determine, or no people in the post); indication that a GLP-1 medication was used primarily for weight loss (yes vs no), health conditions for which GLP-1 medications had approval for treatment, or other prevalent comorbidities noted in the caption, hashtag, or audio (diabetes or blood sugar, overweight and obesity, PCOS, fatty liver disease, hypertension or blood pressure, and other—specify; yes vs no); any mention in the post about lifestyle or health-promoting behaviors (eg, physical activity, eating behaviors; yes vs no), positive emotional well-being (eg, mental health, happiness, confidence; yes vs no), and striving for goals (eg, perseverance toward weight, health goals; yes vs no); before and after comparisons (yes vs no); mentions of clinical side effects (eg, nausea, gastric issues; yes vs no), mentions of nonclinical side effects (eg, “Ozempic face,” loose skin; yes vs no), or negative realities of use (eg, indefinite use, potential weight gain after a period; yes vs no); and overall sentiment toward GLP-1 medications (unfavorable, favorable, or unknown).

For EO 2 related to services and sales of GLP-1 medications, we assessed the following domains: whether the post was offering a service where one could obtain GLP-1 medications (yes vs no) and whether the post included information on where to get information about GLP-1 medications (no; yes, from somewhere perceived to be a traditional health care setting; and yes, any place perceived to be outside of a traditional health care setting).

Finally, for EO 3 related to equity and access, we assessed posts for the following characteristics: observed race or ethnicity of user (Hispanic or Latino, Black or African American, White, Asian, Pacific Islander or Native Hawaiian, Native American or Alaska Native; yes vs no) and any mention of barriers to access including shortages (yes vs no), medications covered by insurance (yes vs no), and out-of-pocket costs (yes vs no).

The first and senior authors developed the initial codebook. To ensure consistency between coders, we used a multistage training and applied learning process. We first held a training session with the research assistant coders (n=5, 100%) that included reviewing the initial codebook as a group, explaining what each code was meant to capture, and providing instructions for how to apply the codes to Instagram postings. During the training, team members asked questions and provided suggestions for improving the clarity of the codebook. The group then reviewed 5 relevant posts that were not part of the sample and applied the codebook. Small modifications were made to the codebook to include additional examples and directions to increase consistency between coders. The coders and the first and senior authors then independently assessed 10 posts from the sample using the codebook. Consistency between coders was calculated, and any posts with discrepancies were reviewed, discussed, and final coding decisions were made by consensus. The codebook was further annotated to document coding decisions made by the full team. This process was repeated with 25 additional posts to improve agreement between coders. The remaining posts were coded by at least 2 members of the research team. Agreement between coders ranged from 69.4% to 97.3%, with the vast majority exceeding 80%. Only 4 codes did not meet this threshold: (1) observed gender of the poster (140/186, 75.3%), (2) indication that a GLP-1 medication was used primarily for weight loss (129/186, 69.4%), (3) striving for goals (130/186, 69.9%), and (4) overall sentiment toward GLP-1 medications (131/186, 70.4%). At this final stage, when there were discrepancies between the two coders, the first and senior authors reviewed the post together, discussed the coding, and made final decisions. Thus, final coding reflects either initial agreement between 2 research assistant coders or 1 research assistant coder and both the first and senior authors.

### Analyses

All analyses were conducted using SPSS (version 29; IBM Corp). Descriptive statistics (eg, frequencies, percentages, and ranges) were run for all variables in the study.

## Results

### Sample

During the coding stage, 311 posts were eliminated, leaving a final sample of 239 Instagram posts. Excluded posts were eliminated primarily because they were not relevant to GLP-1 medications (ie, the post included a study hashtag such as #health, but nothing else in the post was relevant to GLP-1 medications). Only 5 of 239 posts (2.1%) were advertisements or paid partnerships. The final sample of 239 study posts received a total of 162,453 likes, 7971 comments, and 2,581,578 views.

### EO 1: Posts Will be Dominated by Female-Presenting Instagram Users and Contain References Specifically Related to Women’s Physical and Mental Health

As seen in Table 1, of the 239 posts included in the study, 130 (54.4%) posts in the sample were by GLP-1 medication users. More than half of the posts (69/130, 53.1%) suggested that GLP-1 medications were being used specifically for weight loss purposes. Only a small number of GLP-1 medication user posts explicitly named one or more health conditions in their caption, picture, or audio. The most common health condition cited was PCOS (25/130, 19.2%), followed by diabetes (20/130, 15.4%), obesity (11/130, 8.5%), and mental health or disordered eating (9/130, 6.9%). At least 1 post mentioned hypertension or blood pressure, arthritis, and fatty liver disease. Postpartum and weight loss surgery (vertical sleeve gastrectomy and gastric bypass) frequently appeared in posts (not shown in Table 1); however, these were determined to be outside our definition of “health conditions” because they describe the period following childbirth and concurrent or alternative treatment options for overweight and obesity. Almost half of the posts included captions, hashtags, or audio related to lifestyle behaviors (56/130, 43.1%) and sentiments about positive well-being (64/130, 49.2%). Depictions of striving for goals, including health and weight loss journeys, were observed in more than two-thirds of posts (90/130, 69.2%). Less than half of the posts (52/130, 40%) included before and after (or during) comparisons either with photos of the person’s body or with weights (ie, starting vs current weight). Topics that addressed concerns or drawbacks of use were found in a small number of posts, including clinical side effects (4/130, 3.1%), nonclinical side effects (7/130, 5.4%), negative realities of use (2/130, 1.5%), and unknown long-term impact of use (1/130, 0.8%). Instead, the vast majority (100/130, 76.9%) of user posts were considered to include an overall positive sentiment toward GLP-1 medications and their use.

**Table 1:** Description of Instagram posts related to GLP-1^a^ medications (N=239).

Expected observations				Participants
**Expected observation 1: posts will be dominated by female-presenting Instagram users and contain references specifically related to women’s physical and mental health^b^**				
	Observed female^c^ n (%)		90 (92.8)	
	GLP-1 medication use primarily for weight loss (no health condition noted); n (%)		69 (53.1)	
	**Health conditions noted, n (%)**			
		PCOS^d^ or autoimmune conditions	25 (19.2)	
		Diabetes or blood sugar	20 (15.4)	
		Obesity	11 (8.5)	
		Mental health or disordered eating	9 (6.9)	
		Hypertension or blood pressure	2 (1.5)	
		Arthritis	2 (1.5)	
		Fatty liver disease	1 (0.8)	
	Lifestyle behaviors, n (%)		56 (43.1)	
	Positive emotional well-being, n (%)		64 (49.2)	
	Striving for goals (eg, weight loss journey), n (%)		90 (69.2)	
	Before and after images, n (%)		52 (40)	
	Clinical side effects (eg, nausea, gastric issues), n (%)		4 (3.1)	
	Nonclinical side effects (eg, “Ozempic face”), n (%)		7 (5.4)	
	Negative realities of use (eg, indefinite use), n (%)		2 (1.5)	
	Unknown long-term impacts of use, n (%)		1 (0.8)	
	**Sentiments toward medications, n (%)**			
		Favorable	100 (76.9)	
		Unfavorable	2 (1.5)	
		Unknown	28 (21.5)	
**Expected observation 2: posts will offer services to consumers that facilitate access to GLP-1 medications from places perceived to be outside of traditional health care settings (eg, primary care, obesity medicine, endocrinology, or other specialty care)**				
	Offering a service where GLP-1 medications can be obtained, n (%)		55 (23)	
	**Information on how to get GLP-1 medications, n (%)**			
		Traditional health care setting	12 (5)	
		Any place other than traditional health care setting	39 (16.3)	
**Expected observation 3: Posts will address issues related to GLP-1 medication access, including equity in use among marginalized populations, shortages, coverage, and cost**				
	**Observed race or ethnicity of user^b,c,e^ n (%)**			
		Asian	5 (5.2)	
		Black or African American	4 (4.1)	
		Hispanic or Latino	7 (7.2)	
		Native American or Alaska Native	2 (2.1)	
		Pacific Islander or Native Hawaiian	4 (4.1)	
		White	78 (80.4)	
	**Barriers to access, n (%)**			
		Shortages	6 (2.5)	
		Medications not covered by insurance	3 (1.3)	
		Out-of-pocket costs	1 (0.4)	

^a^GLP-1: glucagon-like peptide-1.

^b^Analysis was limited to users of GLP-1 medications (n=130, 54.4 %).

^c^This item was only coded when there was an observable person in the post (n=97, 74.6%).

^d^PCOS: polycystic ovarian syndrome.

^e^Coders could select all that apply.

### EO 2: Posts Will Offer Services to Consumers That Facilitate Access to GLP-1 Medications From Places Perceived to be Outside of Traditional Health Care Settings (eg, Primary Care, Obesity Medicine, Endocrinology, or Other Specialty Care)

Of the 239 posts in the sample, 55 (23%) offered a service to provide GLP-1 medications to consumers. Posts also included information about how or where to get GLP-1 medications. More than 3 times as many posts mentioned getting medications from places that were perceived to be nontraditional health and beauty settings (eg, medical spas and functional medicine providers) as compared with traditional health care settings (39/239, 16.3% and 12/239, 5%, respectively).

### EO 3: Posts Will Address Issues Related to GLP-1 Medication Access, Including Equity in Use Among Marginalized Populations, Shortages, Coverage, and Cost

Findings related to the race and ethnicity of GLP-1 users were limited to only 97 of the 130 (74.6%) user posts where there was an observable person. More than two-thirds of those users (78/97, 80.4%) were perceived to be White, followed by Hispanic or Latino (7/97, 7.2%), Asian (5/97, 5.2%), Pacific Islander or Native Hawaiian (4/97, 4.1%), Black or African American (4/97, 4.1%), and Native American or Alaska Native (2/97, 2.1%). All 239 posts, regardless of user status, were assessed for references about barriers to access. Very few of the posts in the sample noted shortages (6/239, 2.5%), medications not being covered by insurance (3/239, 1.3%), or out-of-pocket costs (1/239, 0.4%).

## Discussion

### Principal Findings

Interest in GLP-1 medications is growing exponentially [15], although there are very few published studies on the public’s use, understanding, or ability to access these medications. In our study, we explored 3 EOs related to how GLP-1 medications were being discussed on Instagram. We found that posts were dominated by female-presenting content creators and included topics relevant to women’s physical and mental health; most posts either offered a service where GLP-1 medications could be obtained or provided information on how to access GLP-1 medications; and few posts addressed issues related to GLP-1 medication access. Of note, at the time of our study, no extant literature existed on the topic. A study [4] conducted concurrently to ours analyzed 100 TikTok posts with the #Ozempic hashtag. The study found that most videos mentioned using or planning to use Ozempic and weight loss, and a very small number of videos mentioned side effects, medication shortages, and off-label prescription use [4]. While there were similarities, our study was broader in scope as it assessed 11 hashtags including a substantially larger number of posts, used a different social media platform, and evaluated additional content.

Consistent with our expectations, we found that the vast majority of GLP-1 medication user posts were posted by women and about women’s experiences with GLP-1 medications; however, the most common health condition cited in posts was PCOS, which we found to be novel. This is among the few studies to document the pervasiveness of this condition among GLP-1 medication users on social media. Content creators called themselves “PCOS warriors” and used hashtags like #pcostreatment, #pcosmanagement, #pcosproblems, and #pcosmamas as indicators of the online community of women who have come together virtually to support each other and share experiences living with an underresearched and difficult-to-manage disease that impacts women specifically [29]. While more research is needed to further explore gender differences on how social media is used to obtain medical information and social support, similar trends have been noted on social media platforms regarding various social and health issues that disproportionately impact women including domestic violence during the COVID-19 pandemic lockdown [30]; breast cancer [31]; and pregnancy, birth, and early parenting experiences such as breastfeeding [32,33].

Given the challenges of PCOS diagnoses and the limited information and treatment options for PCOS, researchers and clinicians alike have found dissatisfaction among their female patients who have been diagnosed with PCOS [34]. Furthermore, as recently highlighted in a study by Keating and Wild [35], the increased interest in GLP-1 medications among women was likely fueled by the lack of appropriate and effective care for women, especially those diagnosed with PCOS—the most prevalent endocrinopathy among women of reproductive age, impacting 6% to 20% of women [22,36]. Our findings are supported by Keating and Wild [35]; they found that #ozempic received more than 500 million views on TikTok (as of February 2023), with a substantial proportion of the videos directly referencing the use of GLP-1 medications for PCOS. Women’s frustrations and desire for more information about PCOS might be leading them to seek information online to educate and advocate for themselves [37]. Given that the number of female adolescents aged 12 to 17 years using GLP-1 medications increased 588% between 2020 and 2023 and 659% for female adults aged 18 to 25 years and that women accounted for 76% of those aged 18 to 25 years who were prescribed a GLP-1 medication in 2023, additional research is needed to determine the impact of these medicines on women’s health (eg, pregnancy, breastfeeding, birth control, and endometriosis) [38].

Furthermore, posts that were included in our study focused nearly entirely on the positive aspects of GLP-1 medication use. This was surprising given the growing attention from various media sources and social media influencers about the adverse effects of GLP-1 medications; we had anticipated that more posts would be dedicated to discussing clinical side effects (eg, nausea), the negative realities of use (eg, indefinite use), or fear around the unknown long-term effects of GLP-1 medications. However, this might be due in part to the hashtags we used in our data collection processes. Our reliance on popular hashtags might have overlooked emerging or niche hashtags, which could have provided different perspectives or insights if included. Future research using the methodology outlined within this paper should include a wider variety of hashtags to ensure a more diverse sample of opinions is included. Also important to consider was the “positivity bias” among social media users, a phenomenon of users limiting posts to favorable or positive aspects of their lives—including framing negative facets of their lives positively for themselves and their followers [39]. Additional efforts should be taken to combat this bias, specifically in the hashtag identification and data collection phases of a study (eg, including hashtags with negative phrases or experiences related to the topic being studied). Ultimately, efforts to continue monitoring GLP-1 medication users’ clinical and nonclinical experiences with the medications were important using a variety of methods, as more people are using the drug and are continuing use for longer periods [40].

As expected, there were a multitude of posts dedicated to selling and promoting GLP-1 medications, both from places that were perceived to be traditional health care settings and nontraditional health and beauty settings (eg, app-based telemedicine, online boutique weight loss medical practices, and medical spas). The legitimacy of the nontraditional health care settings was concerning, given the growing market for off-label products and compounded versions of GLP-1 medications [15,41]. As noted by Basch et al [4], consumers might find it difficult to distinguish between information presented in individuals’ anecdotes versus information that is based on up-to-date and scientific evidence. In the context of media literacy and its impact on health, the growing demand for GLP-1 medications and GLP-1 medication–like alternatives should be studied with careful consideration, especially given that nontraditional health care outlets that are prescribing GLP-1 medications or alternatives can have insufficient or nonexistent health assessments, irregular follow-ups, and limited patient management [15]. Since 2016, the FDA has received an increasing number of complaints from health care providers and consumers about false and misleading prescription drug advertisements on social media platforms [42]. That, in combination with GLP-1 medication shortages, has created an incredible demand for GLP-1 compounds that are not FDA approved or evaluated for safety, efficacy, or quality. The combined demand for GLP-1 medications and the lack of regulatory oversight poses significant risks for users, including significant adverse effects (eg, suicidality, inflammation of the gallbladder, and hospitalization) and prescribing and preparation errors (eg, inappropriate dosing and dosing errors) [43,44]. While TikTok recently developed a policy to reduce the number of GLP-1 medication posts a user on their platform would be exposed to on their “For You” page, mirroring concerns of predatory or dishonest business practices on the public [45], it is increasingly important for social media and endeavored GLP-1 medication users to use caution and connect with a trusted and licensed health care provider before starting a new medicine regiment. The increasing reliance on online pharmacies and social media platforms for access to GLP-1 and GLP-1–like medications highlights the need for additional safeguards to ensure consumer health and safety [46].

The use of GLP-1 medications for treating health issues outside of diabetes and obesity continues to grow—raising concerns about medication shortages and access. For example, in March 2024, the FDA expanded its approval of semaglutide and tirzepatide medications to treat and prevent cardiovascular disease [47]. In addition, GLP-1 medications are being considered for the treatment of sleep apnea, substance use disorders, neurodegenerative disorders (eg, Parkinson disease and Alzheimer disease), insulin resistance, mental health (eg, anxiety and depression), and other diseases (eg, metabolic dysfunction–associated steatohepatitis with liver fibrosis—formerly referred to as fatty liver disease) [48-50]. Medication shortages have been well-documented and have contributed to the growing market for off-label and compounded versions of GLP-1 medications [46]. Although we saw only a small proportion of posts mention issues related to barriers to access in our study (eg, shortages and medications not covered by insurance), given the variety of physical and mental health conditions GLP-1 medications have the potential to solve or alleviate, it is important to critically examine issues around accessing these medications.

Furthermore, and as highlighted by Whitley et al [51], supply issues in 2022 and 2023 significantly disrupted patients’ ability to continue accessing GLP-1 medications and hindered patients’ ability to initiate treatment. Given the health inequalities that exist among those diagnosed with chronic health conditions [52], it is likely that discrimination and biases within our health care system will impact GLP-1 medication prescriptions and access. In recent studies by Eberly et al [53] and Waldrop et al [54], men were less likely than women to be treated with a GLP-1 medication and Black and Latino patients with diabetes were 19% and 9%, respectively, less likely to be treated with a GLP-1 medication as compared with their White counterparts. This was highlighted in our own findings, where most of the users in our sample were observed to be White and female presenting. Health care providers and insurance companies who promote and prescribe GLP-1 medications should continue to carefully address discrimination and biases within their practices and policies to ensure that equitable care is given to their patients with chronic health conditions, regardless of race or ethnicity, gender, or socioeconomic status.

### Limitations

Although this study is novel and is among the first to characterize Instagram posts about GLP-1 medications, there are some limitations worth noting. First, our study included only a limited sample of posts over a 1-week period. These posts might not fully capture the landscape of GLP-1 medication content on Instagram. Future studies could use a longitudinal approach to better capture changes in discourse over time. Furthermore, if researchers are interested in drawing conclusions about the entire population of GLP-1 medication users and posts, they might want to consider in-depth investigations that estimate the incidence of specific posts and the proportions of subcategories. Second, our findings might have limited generalizability in that our sample of posts were largely from and about creators who were observed to be White and female. This study should be replicated using other social media platforms that focus on men and individuals of non-white backgrounds, including those who are Black, Indigenous, and People of Color (BIPOC) to fully understand the landscape of GLP-1 medication content on social media. Third, our measures of gender and race or ethnicity were based on coder observation (ie, visual cues provided within each post); this methodology inherently included risk for misclassification and might not be accurate. We thought the value of exploring women’s health, as well as equity and access, were important enough to use imperfect measures, but recognized that they might have introduced bias into our findings. Similarly, our findings were sensitive to the study coders and how they interpreted what they viewed; this was particularly important given the subjective nature of some of the codes. Finally, our selection of hashtags to use in the study might have influenced our findings (eg, limited appearances of issues related to clinical side effects, trepidation about use, and barriers to access). Future studies should include additional emerging and niche hashtags to ensure that a full range of perspectives and insights are captured. Hashtags that include combinations of terms (eg, #Ozempic AND #access or #disparities or #sideeffects) should also be considered to more deeply explore different themes and trends among GLP-1 medication users and the general social media discourse around GLP-1 medications.

### Conclusions

Overall, posts about GLP-1 medications were largely positive and reflected a growing interest in the use and prescription of GLP-1 medications, although we remain concerned about the availability of medications from nontraditional health care settings and barriers to access among those most at risk. There are clearly potential benefits to individuals who seek information on social media about GLP-1 medications, including increased knowledge, improved perceptions about the medications, and community with others who did or would benefit from GLP-1 medications. Yet, there are legitimate concerns about health and social media literacy of the typical social media user and their ability to adequately assess the accuracy of online health information. The goal should be to have unfettered access to information available for those who seek it; however, the onus is on social media corporations to ensure that online content is accurate and not harmful. Social media companies should find ways to promote content from credentialed content creators, while posts that are potentially harmful or contain misinformation should be labeled or removed. Furthermore, social media companies should consider examining ways in which they can promote social media literacy among users to protect the health and safety of those that were consuming content on their platforms.

Actionable ideas for social media companies are vast but could include creating and implementing a “verified” emblem or “digital identity wallet” for health advertisers (eg, an emblem similar to the blue checkmark currently used by Twitter and Instagram to confirm an account is an authentic public figure or brand, paired with data that include educational degrees or certificates). This would require health care providers who advertise or promote medication on their pages to undergo a verification process to ensure appropriate licensure and compliance with local and national regulations. Criticisms directed toward social media platforms for providing insufficient information about sources and credibility were widespread; however, few studies assessing credential verification exist and should be extended specifically for those who seek health-related information via social media [55,56] to determine if providing sufficient information about the source of health claims could help users make more accurate judgments and assess credibility. As an extension to this suggestion, social media companies could mandate clear disclosure requirements for posters or embed health literacy prompts and warnings in posts. Specifically, disclosures should be required when medications are promoted via paid partnerships or affiliate links—including a statement if the service or medication is being offered by a nonlicensed medical provider.

The health care system also had a role to play. Programs and policies should continue to be implemented to increase equity in the diagnosis and treatment (including prescriptions) for those with chronic diseases. This was especially important to consider given the expansion of the use of GLP-1 medications in treating multiple health issues beyond type 2 diabetes and obesity—especially for women’s health, which had historically been understudied and underfunded [57]. One suggestion would be for medical groups and insurance companies to fund health navigators whose primary role would be to help members navigate insurance and cost barriers to GLP-1 medications (eg, assist patients in how to access manufacturer savings programs, how to request and submit prior authorization documents, and how to write an appeal for medication denials or requests for step-therapies). Drug companies must also get more involved, including addressing the continued shortages of GLP-1 medications, which have encouraged counterfeit and compounded versions of these drugs to infiltrate the market. Furthermore, the price of these medications must be lowered; even though these medications could cost under a dollar to produce, the average cost of a GLP-1 medication without insurance is over US $1000 monthly [58]. Insurance companies that primarily serve low-income and historically marginalized populations, including federal health insurance such as Medicare and Medicaid, have the power to determine whether these medications are covered and who can access them. There should be policy reforms to ensure the coverage of these medications for those with chronic diseases, including overweight and obesity. Ultimately, addressing these concerns would lead to a more knowledgeable and healthy population with equitable access to care.

## Abbreviations

EO: expected observation
FDA: Food and Drug Administration
GLP-1: glucagon-like peptide-1
PCOS: polycystic ovarian syndrome
